# Towards Polymeric Nanoparticles with Multiple Magnetic Patches

**DOI:** 10.3390/nano11010147

**Published:** 2021-01-09

**Authors:** Elham Yammine, Laurent Adumeau, Maher Abboud, Stéphane Mornet, Michel Nakhl, Etienne Duguet

**Affiliations:** 1 Univ. Bordeaux, CNRS, Bordeaux INP, ICMCB, UMR 5026, 33600 Pessac, France; elhamyammine93@gmail.com (E.Y.); laurent.adumeau@cbni.ucd.ie (L.A.); stephane.mornet@icmcb.cnrs.fr (S.M.); 2 LCPM/PR2N (EDST), Lebanese University, Faculty of Sciences II, Jdeidet El Metn 90656, Lebanon; mnakhl@ul.edu.lb; 3 Unité Environnement Génomique et Protéomique, U-EGP, Faculté des Sciences, Université Saint-Joseph, Campus des Sciences et Technologies Mar Roukos–B.P. 1514, Riad El Solh, Beirut 1107 2050, Lebanon; maher.abboud@usj.edu.lb

**Keywords:** silica, polystyrene, maghemite supraparticles, patchy particles, seeded-growth emulsion polymerization, solvent-induced self-assembly

## Abstract

Fabricating future materials by self-assembly of nano-building blocks programmed to generate specific lattices is among the most challenging goals of nanotechnology and has led to the recent concept of patchy particles. We report here a simple strategy to fabricate polystyrene nanoparticles with several silica patches based on the solvent-induced self-assembly of silica/polystyrene monopods. The latter are obtained with morphological yields as high as 99% by seed-growth emulsion polymerization of styrene in the presence of 100 nm silica seeds previously modified with an optimal surface density of methacryloxymethyl groups. In addition, we fabricate “magnetic” silica seeds by silica encapsulation of preformed maghemite supraparticles. The polystyrene pod, i.e., surface nodule, serves as a sticky point when the monopods are incubated in a bad/good solvent mixture for polystyrene, e.g., ethanol/tetrahydrofuran mixtures. After self-assembly, mixtures of particles with two, three, four silica or magnetic silica patches are mainly obtained. The influence of experimental parameters such as the ethanol/tetrahydrofuran volume ratio, monopod concentration and incubation time is studied. Further developments would consist of obtaining pure batches by centrifugal sorting and optimizing the relative position of the patches in conventional repulsion figures.

## 1. Introduction

Building hierarchical structures with unique properties for both fundamental studies and technological applications is among the most active research areas in nanotechnology. For bottom-up approaches, the main current goal is the preparation of nanoparticles serving as building blocks for controlled assembly [1]. However, isotropic spherical nanoparticles lead to only a low number of close-packed colloidal lattices, while anisotropic nanoparticles are able to order into many novel crystalline or non-crystalline structures [2]. In this context, there is great interest in patchy particles envisioned as valence-endowed colloidal atoms for targeting specifically more open lattices and low-coordination architectures [3,4,5,6,7,8]. These surface-patterned particles are indeed designed to experience specific directional interactions with neighboring particles. Among the investigated attractive forces, magnetic dipolar interactions have received specific attention over the past fifteen years [9,10,11,12,13,14,15]. Particles with a single magnetic patch were essentially investigated, because the dipole moment is shifted out of the particle center, and original assemblies were obtained according to the predictions of simulation studies. These monopatch particles are rather easily fabricated, on a wide range of scales from a few hundred nanometers to a few millimeters, through conventional dissymmetrisation routes used to produce Janus particles [15]. Previous studies have neglected the option of particles carrying multiple magnetic patches. The difficulty of manufacturing such objects is not the only problem, since to our knowledge, the simulators have not published such studies either, while studies of particle assembly having several nonmagnetic patches abound [8]. Nevertheless, the possibility of making particles with two magnetic patches was reported twice. A microfluidic route using double emulsion templates encapsulated two droplets of magnetic particles in hydrogel microparticles by increasing the inner flow [16]. Two-patch particles were also reported as by-products when manufacturing organosilica microparticles carrying one hematite cube [17]. In both cases, the relative position of the two patches was not finely tuned, and there is, therefore, a need to find synthesis routes of particles having 2, 3, 4, etc. magnetic patches, if possible, to position these according to predefined geometric figures.

The paradigm shift that we propose here is to consider the synthesis of particles with several magnetic patches through the self-assembly of monopatch particles by fusing their nonmagnetic components. It concerns polymer-based particles, and we drew inspiration from some reports that relate to nonmagnetic colloids. Micron-sized asymmetric dumbbells made of a polystyrene (PS) sphere and a poly(n-butylacrylate) lobe self-assemble into well-defined clusters when dispersed in aqueous media, when the desorption of the poly(vinylpyrrolidone) stabilizer from the particles triggers the merging of the soft poly(n-butylacrylate) lobes upon contact through collision [18]. Later, other researchers fabricated by a microfluidic technology 200 µm hydrophilic poly(ethylene glycol) diacrylate particles with a 160 µm ethoxylated trimethylolpropane tri-acrylate lobe [19]. They obtained quite regular submillimeter-sized clusters by drying Pickering-like water droplets stabilized from these particles. More recently, we reported that dumbbell-shaped silica/PS nanoparticles can self-assemble in regular clusters by incubation in mixtures of bad/good solvents for PS, i.e., ethanol/dimethylformamide (DMF) mixtures [20]. In such conditions, the partial swelling of the PS patch makes it sticky enough to attract and merge to the PS patches of other particles.

**Figure 1:**
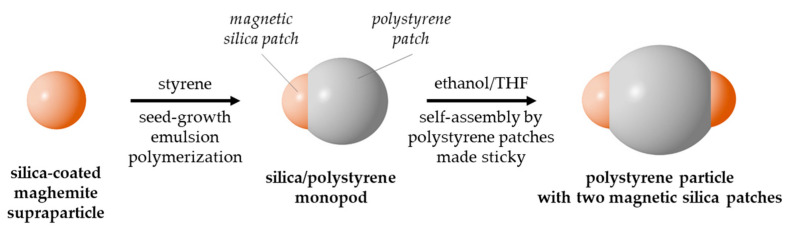
Scheme showing the two-step synthesis pathway to obtain polystyrene particles with several magnetic silica patches, exemplified here for two patches.

In the present study, we extent this concept to magnetic silica/PS asymmetric nanoparticles in order to obtain PS particles bearing several patches made of “magnetic” silica, i.e., silica nanoparticle encapsulation of a supraparticle obtained by superparamagnetic maghemite nanocrystal close-packing. As shown in Figure 1, the first step consists in the dissymmetrisation of the magnetic silica nanoparticle by a PS nodule obtained after seed-growth emulsion polymerization of styrene. This silica/PS monopod can in fact be described twice as a monopatch particle depending on whether we consider the PS nodule, i.e., PS patch, which will be used for solvent-induced self-assembly (2nd step), or the magnetic silica particle, i.e., magnetic patch, whose forthcoming studies will take benefit for subsequent assembly assisted by magnetic fields. Our strategy consisted of (i) optimizing the operating conditions of the two steps with model silica particles, (ii) finding an indisputable way to check that the silica patches protrude out of the surface of the PS particles throughout the process and (iii) showing that the process is robust enough to work for magnetic silica patches. Our results are essentially supported by statistical studies carried out using transmission electron microscopy (TEM) images.

## 2. Materials and Methods

### 2.1. Materials

Tetraethoxysilane (TEOS, ≥99%), styrene (≥99%, with ca. 50 ppm 4-tert-butylcatechol as stabilizer), sodium persulfate (Na_2_S_2_O_8_, ≥99%), polyethylene glycol nonylphenyl ether (Synperonic^®^ NP30), sodium dodecylsulfate (SDS, 99%), hydroquinone (≥99%), cyclohexane (≥99.7%) and tetrahydrofuran (THF, ≥99% contains 250 ppm butylhydroxytoluene as inhibitor) were purchased from Sigma-Aldrich (Saint-Quentin Fallavier, France). 3-methacryloxypropyl(trimethoxy)silane (MPS, 98%) was purchased from ABCR (Karlsruhe, Germany). Ammonium hydroxide (NH_4_OH, 28–30% in water) and ethanol (99%) were provided by Atlantic Labo (Bruges, France). Oleic acid (65–88%) was purchased from Fisher Scientific (Illkirch-Graffenstaden, France). Deionized water with a resistivity of 18.2 MΩ·cm at 25 °C was obtained from a Milli-Q system (Merck Millipore, Darmstadt, Germany). All chemicals were used without further purification.

### 2.2. Colloid Synthesis

Silica nanoparticles with an average diameter of 100 nm and polydispersity index of 1.003 were obtained by TEOS hydrolysis/polycondensation according to an already published protocol [21]. At the end of the synthesis, the silica surface was functionalized with methacryloxypropyl functions by reacting with MPS at room temperature for 3 h and then one more hour at 90 °C under stirring. The MPS volume (V_MPS_) was calculated for tuning the nominal grafting surface density (d_MPS_) between 0.3 and 2 funct./nm^2^ according to Equation (1):

(1) $$V_{\mathrm{MPS}}=\frac{6\cdot {\;d}_{\mathrm{MPS}}\cdot {\;M}_{\mathrm{MPS}}\cdot {\;V}_{\mathrm{silica}}\cdot {\;N}_{\mathrm{silica}}\;}{N_{A}\cdot \rho_{\mathrm{MPS}}\cdot \rho_{\mathrm{silica}}\cdot D_{\mathrm{silica}}}$$

where N, M, D, N_A_ and ρ symbolize particle number, molar mass, particle diameter, Avogadro number and density, respectively. After dialysis against MilliQ water, the mass concentration of silica particles was checked by the dry-extract method.

Magnetic silica nanoparticles were prepared by silica strengthening and coating of maghemite supraparticles whose synthesis by the emulsion evaporation route using SDS as surfactant was previously reported by Simard and coworkers [22]. For this purpose, we used a hydrophobic ferrofluid synthesized from 7.5 nm-sized maghemite nanoparticles prepared in a conventional manner by alkaline coprecipitation [23] and stabilized by oleic acid in cyclohexane according to a protocol previously reported by van Ewijk et al. [24]. We describe here in detail only the silica strengthening and coating stages. First, 250 mg of 79 ± 10 nm-sized maghemite supraparticles dispersed in 20 mL SDS aqueous solution (50 mM) were diluted with 30 mL water, before adding successively 2.5 mL NH_4_OH and 250 μL TEOS. After stirring for 2 h at 40 °C, 200 mL water was added, before two centrifugation stages: a first one (8000× *g*; 15 min) to recover the pellet and redispersion in water and a second (500× *g*; 15 min) to recover the supernatant. The final volume of the dispersion was adjusted to 20 mL with water. Secondly, we added 72 mL ethanol, 4.4 mL ammonia and then a known volume of TEOS (0.1 mL/h). The reaction medium was stirred at room temperature for at least 4 h. The ammonia and some of the ethanol were removed using a rotary evaporator before washing the particles in a water/ethanol mixture (1:1) by two centrifugation/redispersion cycles (10,000× *g*; 15 min). The TEOS volume was calculated as a function of the targeted diameter D of the magnetic silica nanoparticles from the initial average diameter d of the maghemite supraparticles according to Equation (2):

(2) $$V_{\mathrm{TEOS}}=\;\frac{\pi \cdot \rho_{{\mathrm{SiO}}_{2}}\cdot M_{\mathrm{TEOS}}}{6\cdot M_{{\mathrm{SiO}}_{2}}\cdot \rho_{\mathrm{TEOS}}}\cdot N_{{\mathrm{Fe}}_{2}O_{3}\;\mathrm{supraparticle}}\cdot \left(D^{3}-d^{3}\right)$$

Nevertheless, as shown in Section 3.3, Equation (2) underestimates the amount of TEOS serving to fill the space between the 7.5 nm maghemite nanoparticles within the supraparticles. Therefore, future researchers are invited to use instead Equation (3), which includes further a corrective term considering a random close packing density of 0.64 for the maghemite nanoparticles inside each supraparticle [25]:

(3) $$V_{\mathrm{TEOS}}=\;\frac{\pi \cdot \rho_{{\mathrm{SiO}}_{2}}\cdot M_{\mathrm{TEOS}}}{6\cdot M_{{\mathrm{SiO}}_{2}}\cdot \rho_{\mathrm{TEOS}}}\cdot N_{{\mathrm{Fe}}_{2}O_{3}\;\mathrm{supraparticle}}\cdot \left[\left(D^{3}-d^{3}\right)+0.36{\;d}^{3}\right]$$

For this study, magnetic silica seeds with a diameter of 105 ± 10 nm were obtained corresponding to a silica thickness of 13 nm. The maghemite weight fraction was estimated to be about 48% using Equation (4):

(4) $$\mathrm{wt}.\;\%\left({\mathrm{Fe}}_{2}O_{3}\right)=\;\frac{0.64{\;d}^{3}\cdot \rho_{{\mathrm{Fe}}_{2}O_{3}}}{\left[\left(D^{3}-d^{3}\right)+0.36{\;d}^{3}\right]\cdot \rho_{{\mathrm{SiO}}_{2}}+0.64{\;d}^{3}\cdot \rho_{{\mathrm{Fe}}_{2}O_{3}}}\cdot \;100$$

The silica/PS monopods were obtained through seed-growth emulsion polymerization of styrene, according to a first protocol already fully described in our previous publications [26]. Briefly, 50 mL emulsion was prepared by mixing Synperonic^®^ NP30 (2.85 g/L), SDS (0.15 g/L), the MPS-modified silica nanoparticles (7.3 × 10^15^ part./L), styrene (89 g/L) and Na_2_S_2_O_8_ (0.46 g/L) in MilliQ water. Then, the polymerization was triggered by increasing the temperature to 70 °C for 6 h. Monomer-to-polymer conversion was determined by the dry-extract method and comprised between 60% and 95%. When necessary, free PS particles were removed by discarding supernatant after two centrifugation cycles (2000 g; 15 min) after dilution 10 times in water or ethanol. The monopods were redispersed in water or ethanol, and the dispersion concentration was determined by the dry-extract method.

An alternative emulsion polymerization protocol was also used, inspired by the work of Ge et al. [27], and especially well adapted for low seed concentrations. Briefly, a 15 mL emulsion was prepared by mixing Synperonic^®^ NP30 (0.19 g/L), SDS (0.01 g/L), the MPS-modified silica nanoparticles (4.8 × 10^14^ part./L), styrene (56.8 g/L) and Na_2_S_2_O_8_ (0.78 g/L) in MilliQ water. The polymerization temperature and time employed were identical to those of the previous protocol.

### 2.3. Solvent-Induced Colloidal Self-Assembly

We were inspired by our own work concerning the assembly of silica/PS monopods but displaying another morphology [20]. The self-assembly of each type of monopod was carried out in a 2.5 mL glass bottle closed with a polypropylene cap, gently stirred on a roller-mixer at 60 rpm. The quantities of monopod (dispersed in water or ethanol), of good (DMF or THF) and bad solvent (water or ethanol), for PS were carefully measured to reach a final volume of 1 mL.

### 2.4. Characterization by Electron Microscopy

The morphology and size of colloids were studied for each batch by transmission electron microscopy (TEM) using a JEM 1400+ LaB_6_ apparatus sourced from JEOL Europe SAS (Croissy-sur-Seine, France) operating at 120 kV. The samples were prepared by directly depositing a drop of the colloid dispersion on the TEM grids. Statistical image analyses were performed over at least 150 nanoparticles. Size polydispersity indexes were calculated from the weight average $\bar{D_{w}}$ and number average $\bar{D_{n}}$ diameter values according to Equation (5):

(5) $$\mathrm{size}\;\mathrm{polydispersity}\;\mathrm{index}=\;\bar{D_{w}}/\bar{D_{n}}=\frac{\sum^{}n_{i}D_{i}^{4}}{\sum^{}n_{i}D_{i}}/\frac{\sum^{}n_{i}D_{i}^{3}}{\sum^{}n_{i}}$$

To check that the surface of the silica was not covered by even a very thin PS layer, we carried out a step of silica regrowth before TEM observation, according to the following operating mode [28]. To 1 mL of the particle dispersion in ethanol or water, we added 45.5 mL ethanol and 3.5 mL NH_4_OH. Then, we added dropwise a solution of 3.8 mL TEOS (10 vol.% in absolute ethanol) using a single-syringe pump (1 mL/h). The particles were washed using two centrifugation/redispersion cycles in ethanol (12,000× *g*; 10 min).

Elemental mapping by scanning transmission electron microscopy coupled to energy dispersive X-ray spectrometry (STEM-EDX) was carried out with a JEM 2200 FS apparatus sourced from JEOL Europe SAS (Croissy-sur-Seine, France) equipped with a field emissive gun operating at 200 kV. The samples were prepared as for conventional TEM experiments.

## 3. Results and Discussion

### 3.1. Preparation of Silica/Polystyrene Monopods

We prepared batches of size-monodisperse silica seeds with a diameter of 100 nm and surface methacryloxypropyl groups at nominal grafting densities from 0.3 to 2 funct./nm^2^. Each one was then used as seeds in an emulsion polymerization process of styrene [21,29] in order to find the optimized conditions to promote nucleation and growth of a single PS nodule on each silica seed. It is worth mentioning that high amounts of free PS particles, i.e., which do not contain a silica core, were also observed in the TEM images (Figure S1). Nevertheless, they may be easily removed by two centrifugation cycles. The features of the as-obtained batches and their statistical morphological analysis from TEM images are summarized in Table 1. The lower the nominal grafting density (entries #1.1 and #1.2), the higher the number of PS nodules (up to 4 and 3, respectively). Therefore, the number of monopods was too low. From 0.6 to 2 funct./nm^2^ (entries #1.3 to #1.6), monopods were systematically obtained with morphological yield as high as 99%. This phenomenon may be explained as follows: the higher the nominal grafting density, the more organophilic the silica surface, the smaller the contact angle of the growing PS nodules on the silica seed and, therefore, the lower their surface mobility and the easier it is for them to merge into a single nodule. This is particularly evidenced by the diameter of the PS nodule of the monopods, which is systematically much higher than that of the free PS particles that have grown independently from the silica seeds.

**Figure 2:**
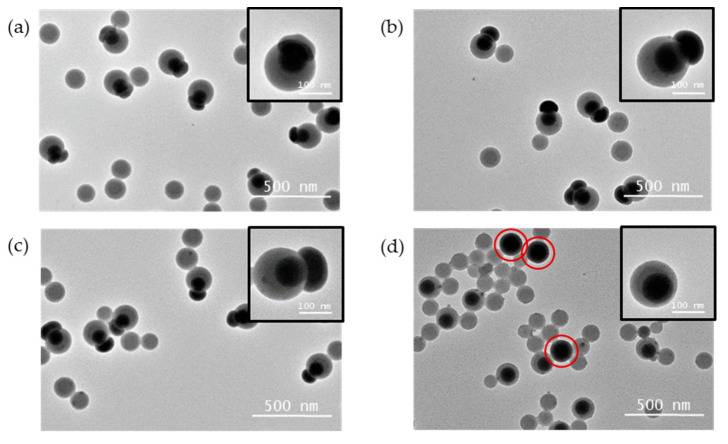
TEM images of silica/PS nanoparticles obtained for different nominal grafting surface densities d_MPS_ and after silica regrowth: (**a**) 0.6 funct./nm^2^; (**b**) 0.7 funct./nm^2^; (**c**) 1 funct./nm^2^; (**d**) 2 funct./nm^2^. The red circles show free silica particles obtained after the silica regrowth. The free PS particles observed in the images result from the polymerization step, because they were not removed by centrifugation for this series of experiments.

As seen in the TEM images (Figure S1), even if the difference in contrast between silica and PS is sufficient to distinguish both phases, the exact particle morphological interpretation is strongly dependent on its orientation on the TEM grid. Indeed, it is not so easy to determine whether the silica core protrudes out of the surface of the PS particle or else it is just below the surface covered at this position by a thin polymer layer. Because this criterion is critical to our end goal of obtaining accessible inorganic patches, we collected 1 mL of each monopod batch (entries #1.3 to #1.6) and added ethanol NH_4_OH and TEOS, i.e., experimental conditions expected to promote the silica core regrowth [28]. Figure 2 shows the TEM images of each batch taken after this treatment. We can observe for the three batches prepared from seeds with 0.6 ≤ d_MPS_ ≤ 1 funct./nm^2^ the presence of a silica protrusion attached to the silica core indicating its accessibility to the reagents (Figure 2a–c). However, this was not true for the last batch (d_MPS_ = 2 funct./nm^2^): new silica particles, circled in red in Figure 2d, did appear but separately from the silica/PS nanoparticles. Therefore, the latter is not composed of monopods as expected, because the silica core is fully encapsulated in the PS particle. Such decentered core-shell particles result from a degree of PS on silica wettability that is too high, as reported by Reculusa et al. [30] and Lang and coworkers [31,32] under quite similar conditions.

To further understand the mechanism of monopod formation during the seeded emulsion polymerization, we collected samples over time, taking care to immediately deactivate the polymerization reaction by adding hydroquinone as inhibitor, and storing the samples at 4 °C. Each sample was observed by TEM to perform statistical analyses of the morphological evolution.

**Figure 3:**
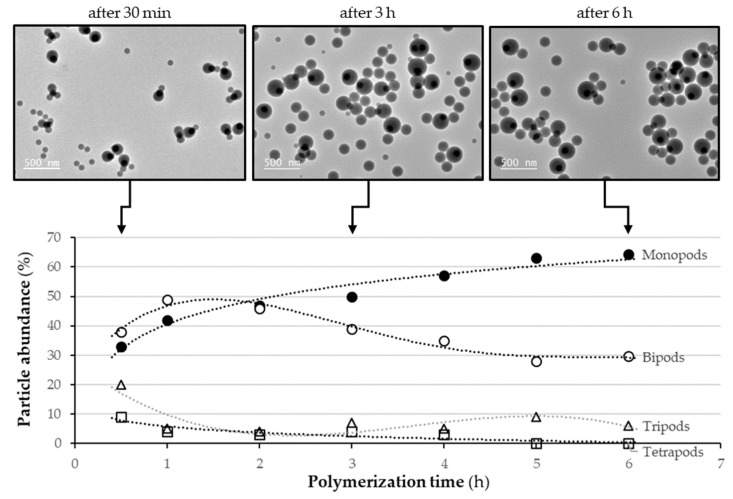
Graph showing the evolution with polymerization time of the abundance of the four main types of silica/PS nanoparticles as observed by TEM from silica seeds with d_MPS_ = 0.5 funct./nm^2^, and representative TEM images taken after 30 min, 3 h and 6 h. Experimental conditions: [100-nm silica] = 7.3 × 10^15^ part./L; [styrene] = 89 g/L; [Synperonic^®^ NP30] = 2.85 g/L; [SDS] = 0.15 g/L; [Na_2_S_2_O_8_] = 0.46 g/L and 70 °C. The dash-dotted curves are a guide for the eye.

We first studied the polymerization experiment carried out on seeds with d_MPS_ = 0.5 funct./nm^2^ (Figure 3). We observed that after 30 min, monopods, bipods, tripods and tetrapods coexisted and that bipods were the main population. As the polymerization progressed, the size of the PS nodules increased, and the tetrapods, tripods and bipods gradually disappeared in favor of the monopods whose abundance increased from 33% to 63% after 6 h. This growth mechanism is consistent with Thill’s model that we reported a few years ago [29,33]. This means that the nucleation of PS particles leads to the coexistence of several PS nodules on the silica seed. As they grow, i.e., as the seed surface is increasingly crowded, they can move, coalesce and/or leave the seed. Here, coalescence seemed to be the preferred scenario because the proportion of free particles appeared to be constant and the bipods after 6 h were dissymmetric with both nodules of different size. Thill’s model also makes it possible to explain the non-negligible quantity of bipods, which remain at the end of the polymerization. Indeed, when there are only two nodules of PS left on the surface, the coalescence probability is almost zero if they are positioned at an angle of 180°, because they can thus grow to an infinite size independently. Thus, this growth mechanism is not favorable to obtain pure batches of monopods.

**Figure 4:**
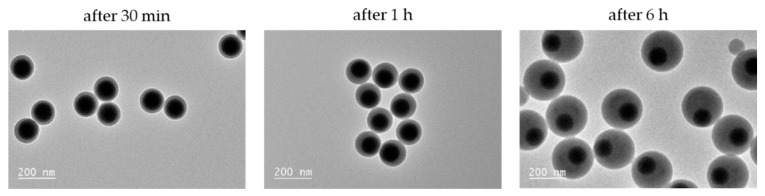
TEM images showing the evolution with time of the morphology of the silica/PS monopods obtained from silica seeds with d_MPS_ = 0.6 funct./nm^2^. Experimental conditions: [100-nm silica] = 7.3 × 10^15^ part./L; [styrene] = 89 g/L; [Synperonic^®^ NP30] = 2.85 g/L; [SDS] = 0.15 g/L; [Na_2_S_2_O_8_] = 0.46 g/L and 70 °C. Before observation, the free PS particles were removed by one centrifugation/redispersion cycle in ethanol (2000× *g*; 15 min).

When we studied the growth mechanism from silica seeds with d_MPS_ = 0.6 funct./nm^2^, we observed a different process of monopod formation (Figure 4): after 30 min of polymerization, the reactor contained exclusively one type of PS/silica nanoparticle with one PS nodule which almost entirely wrapped around the silica core. As the polymerization time and consequently the polymer nodule size increased, this very low contact angle was maintained and led, after 6 h, to a monopod yield of 99%, as already mentioned in Table 1, entry #1.3.

Therefore, we concluded the existence of three main regimes for monopod formation as a function of the surface grafting density of the methacryloxypropyl functions on the seed surface, which dictates the contact angle of the PS nodule on the silica core:

- For d_MPS_ ≤ 0.5 funct./nm^2^, the fairly high value of the contact angle leads to the coexistence of several nodules on the surface of the silica seed and the phenomenon of coalescence is insufficient for all the initially generated structures to transform ultimately into monopods;

- For 0.6 ≤ d_MPS_ ≤ 1 funct./nm^2^, the contact angle is sufficiently low to form a single PS nodule, while leaving the silica core partially protruding, i.e., part of the silica surface remains accessible;

- For d_MPS_ ≥ 2 funct./nm^2^, the contact angle is too low and the silica core is engulfed in the polymer nodule giving decentered core-shell nanoparticles.

**Figure 5:**
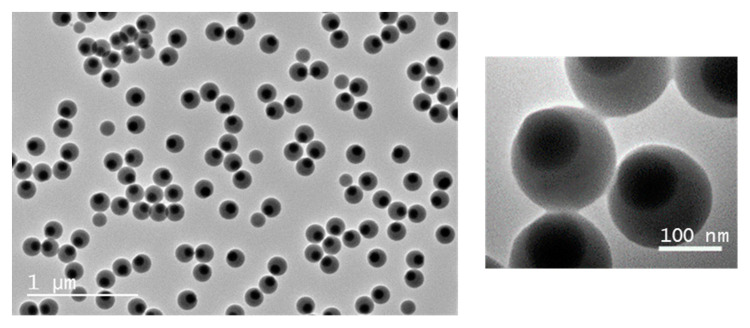
TEM images of silica/PS monopods (Table 1, entry #1.3) after two sorting cycles by centrifugation/redispersion (2000× *g*; 15 min).

For the purpose of our study, only the second growth regime is relevant. To check the repeatability of the experiments, the synthesis of monopods from silica seeds with d_MPS_ = 0.6 funct/nm^2^ (Table 1, entry #1.3) was reproduced twice (Table 1, entries #1.7 and #1.8). Only slight differences were observed in the diameter of the PS nodule consistently with differences measured in the monomer-to-polymer conversion. The self-assembly experiments discussed in the next section were essentially performed with the monopod batch described in Table 1 (entry #1.3). The fraction of free PS particles was easily lowered below 5% thanks to two centrifugation/redispersion cycles (Figure 5).

### 3.2. Solvent-Induced Self-Assembly of Silica/Polystyrene Monopods

We carried out a protocol similar to one we recently reported about differently-shaped monopods [20]. It consists of making the PS patches sticky by dispersing the monopods in a good solvent, e.g., THF or DMF, in order to swell the nodules, without dissolving them entirely. To avoid the dissolution, the quality of the solvent was lowered by the presence of a second liquid, which is both miscible with the good solvent and non-solvent for PS, e.g., water or ethanol. For each experiment, we used 7.3 × 10^11^ monopods in a total liquid volume of 1 mL. The good solvent fraction was introduced last, and the samples were continuously stirred. The self-assembly was investigated by TEM statistical analyses after collection, deposition on the grid and evaporation of a sample microdroplet. By counting the number of silica particles henceforth combined in the same PS particle, we determined the number N of silica patches, meaning that N = 1 for unassembled monopods. N may be also considered here as the aggregation number.

Preliminary trials were performed with the following bad/good solvent systems: ethanol/DMF, water/THF and ethanol/THF mixtures in 20/80 volume ratio. Only the latter system led to significant self-assembly of the monopods. That is why we focused our study on the THF/ethanol system, and we performed a systematic study by varying the volume fraction of THF (Table 2, entries #2.1–#2.5). When the volume fraction of THF was lower than 70%, the amount of good solvent was too low for making the PS nodule sticky enough, and no multipatch particles was observed. From 70%, PS particles with two or more silica patches were observed, but their quantities remained low, resulting from the self-assembly of no more than one fourth of the monopods. Moreover, the higher the THF fraction, the more numerous the tiny and coalescing PS particles resulting from the partial extraction by solubilization and precipitation of PS macromolecules. This is why we decided that a 20/80 ethanol/THF volume ratio is optimal for our experimental conditions. In order to increase the quantities of multipatch PS particles, we increased the concentration of monopods to increase the probability of the particles meeting (Table 2, entries #2.4 and #2.6–#2.7). Indeed, quadruple the concentration made it possible, all other parameters being equal, to increase the assembled fraction of monopods from 20% to 62%. In such conditions, 35% led to two-patch particles, 17% led to three-patch particles and 10% to particles with higher patch numbers.

**Figure 6:**
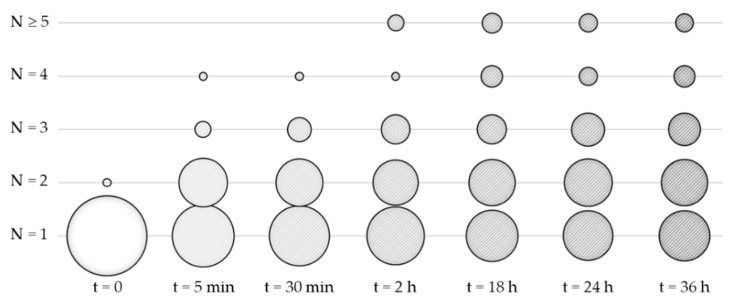
Evolution of the distribution of the number of silica patches with time. The surface of the disks is proportional to the fraction of the monopods assembled in PS particles of similar patch number. Self-assembly experimental conditions: [monopods] = 4C = 2.9 × 10^15^ part./L at 25 °C in ethanol/THF volume ratio 20/80. The data for t = 0 and t = 24 h correspond to the Entry #1.3 of Table 1 and Entry #2.7 of Table 2, respectively.

With the optimized conditions of this last experiment (Table 2, entry #2.7), we studied the kinetics of self-assembly by collecting and analyzing samples over time (Figure 6). It can be observed that within the first minutes, a large number of particles with two patches was formed as well as some others with 3 or 4 patches. Then, these proportions changed more slowly in favor of a progressive increase in the average number of patches, mainly to the detriment of the monopod population. The two-patch particle fraction remained relatively stable. Beyond 18 h, the variations in the composition of the system were of the same order of magnitude as the margin of error in the statistical analysis, which showed that an equilibrium was probably reached. We also studied the impact of an increase in temperature over the 25–45 °C range, and we showed that it has a relatively small influence on the distribution of the number of patches on the PS particles—we observed a slight increase in the average number—but this significantly complicated the experimental setup. Thus, we carried out the next experiments at room temperature.

**Figure 7:**
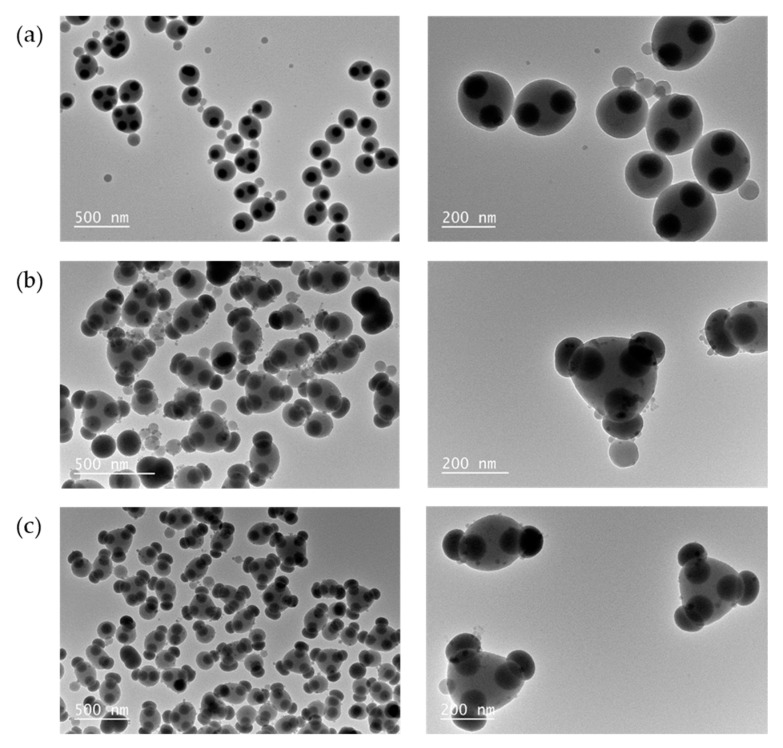
Representative TEM images of (**a**) PS particles with silica patches as obtained according to experimental conditions described in Table 2, entry #2.7; (**b**) the same particles after silica regrowth; and (**c**) the particles obtained after assembly in similar conditions of the silica/PS monopods after silica regrowth.

Typical TEM images of as-obtained PS particles with several silica patches are displayed in Figure 7a. To check that the surface of the silica patches was still accessible, we performed the test of silica regrowth (Figure 7b). We can observe that silica protrusions appeared on the surface of the silica patches proving as previously that they protrude out of the surface of the PS particles. To verify that the self-assembly protocol was robust, we proceeded to assemble the monopods on the surface of which we had previously created a silica protrusion. Figure 7c shows TEM images very similar to those of Figure 7b, which proves on the one hand that the regrowth of the silica can be carried out as well before or after the assembly, and on the other hand that the assembly of the monopods is primarily controlled by the PS patch and not the silica patch. One would have expected that the steric hindrance of the silica protrusion would lead to particles that are more symmetrical. However, the comparison of TEM images shows that the deviations from the repulsion figures, e.g., straight line and equilateral triangle for particles with two and three patches, respectively, were very similar.

### 3.3. Extending the Strategy to Magnetic Silica Patches

To return to our primary objective of making particles with several magnetic patches, we tried to apply the experimental conditions, optimized in the previous sections, using magnetic silica particles thereafter. They are in fact maghemite supraparticles encapsulated in a silica shell so that the final size is around 100 nm, that is, equivalent to those of the model silica patches used earlier. The maghemite supraparticles were prepared through the evaporation-induced emulsion route [22] that we adapted from the original work to replace the polyacrylate-based coating by silica. This multistep procedure includes:

- The preparation of a hydrophobic ferrofluid from 7.5 nm maghemite nanoparticles surface-coated with oleic acid and dispersed in cyclohexane. Compared to the initial protocol [22], cyclohexane was preferred to toluene because its viscosity is slightly higher and makes it possible to minimize the formation of small satellite droplets during droplet rupturing;

- Its emulsification in water in the presence of SDS as surfactant;

- The cyclohexane evaporation to obtain 79 ± 10 nm sized maghemite supraparticles dispersed in water thanks to the outer layer of SDS;

- Their mechanical strengthening with silica which first requires a delicate transfer of the supra-particles in a hydro alcoholic medium—made possible by the addition of SDS—and then is performed in two steps. We showed by TEM analysis of the as-obtained magnetic silica particles that the thickness of the observed silica layer was always about 8 nm less than the expected thickness (Figure S3a). This demonstrates that part of the produced silica serves above all to fill the space between the 7.5 nm maghemite nanoparticles within the supraparticles, as confirmed by infrared absorption spectroscopy analysis, which showed no traces of SDS or oleic acid in the magnetic silica particles (Figure S3b).

Before describing the preparation of the magnetic silica/PS monopods, two notable differences with regard to the pure silica seeds shall be noted. First, the size polydispersity was greater, despite the fact that the size distribution was narrowed by two centrifugation steps, one to remove the smallest and the other to remove the largest. Thus, the diameter of the magnetic silica particles used was 105 ± 10 nm (Figure S3c), i.e., a size polydispersity index of 1.750 against 1.003 for the pure silica seeds. Second, the available quantities of magnetic silica particles were quite low, leading us to adapt the seed-growth emulsion polymerization protocol.

We used the seed-growth emulsion polymerization protocol reported by Yin and coworkers [27], which made it possible to reduce by a factor of 15 the seed concentration. We first checked that these new experimental conditions led to the preparation of monopods from pure silica seeds with d_MPS_ = 0.6 funct./nm^2^ corresponding to the optimal value determined previously for the original polymerization protocol (Table 3, entry #3.0). Then, we used magnetic silica seeds with the same nominal MPS grafting density (Table 3, entry #3.1; Figure S4a). In that case, the observed fraction of monopods was very low (4%), and two thirds of the multipods were undesired hexapods. Therefore, we increased progressively the nominal MPS grafting density and obtained for d_MPS_ = 1.9 funct./nm^2^ essentially monopods with morphological yield as high as 90% for experiments repeated three times under the same conditions (Table 3, entries #3.4 to #3.6). The main side-product was PS particles containing several magnetic silica seeds and/or bipods. Two reasons can explain why we needed a nominal MPS grafting density more than 3 times greater. First, we can not exclude different silica roughness and porosity between the pure seeds and the magnetic seeds; therefore, d_MPS_ was probably underestimated for magnetic seeds. Second, their size polydispersity could also be another source of calculation error.

**Figure 8:**
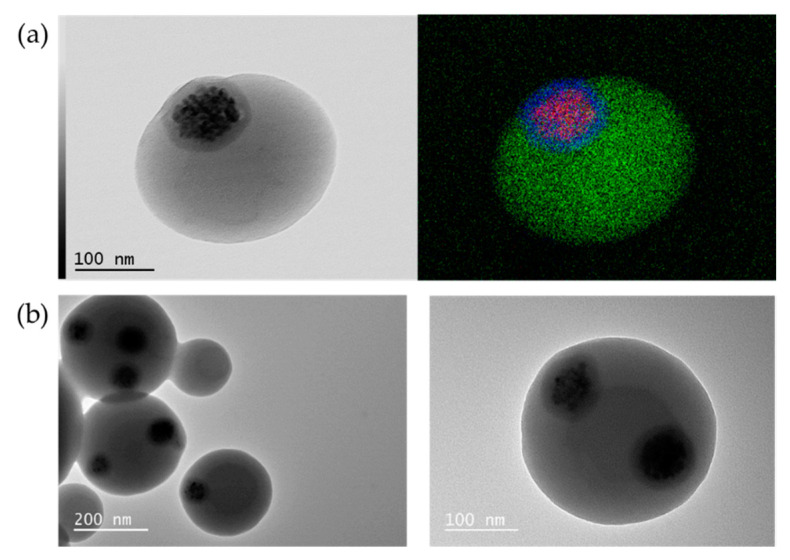
(**a**) STEM image (left) and EDX elemental mapping (right) of a representative magnetic silica/PS monopod (red, blue and green pixels represent iron, silicon and carbon, respectively) corresponding to the entry #3.6 of Table 3; (**b**) TEM images of PS particles with several magnetic patches as obtained after assembly of the magnetic monopods, corresponding to entry #3.6 of Table 3 collected at t = 30 min, using the optimal conditions as defined in entry #2.4 of Table 2.

During the synthesis of the batch of magnetic monopods corresponding to entry #3.6 of Table 3, we collected two samples at t = 30 min and t = 3 h to compare the size of the PS nodule with that obtained at the end of reaction, i.e., for t = 6 h. We then observed an average PS nodule-to-magnetic seed size ratio of 1.6, 2.2 and 2.4, respectively. Moreover, we performed EDX analysis in STEM mode to show the presence of carbon, silicon and iron elements and to confirm the presence of iron oxide nanoparticles within the silica patch while this patch protrudes slightly out of the PS particle (Figure 8a).

Lastly, we performed preliminary experiments of solvent-induced self-assembly with the magnetic monopods corresponding to entry #3.6 of Table 3 collected at t = 30 min, i.e., with an average PS nodule-to-magnetic seed size ratio of 1.6. We used the optimal assembly conditions as defined in entry #2.4 (Table 2). After 24 h, we observed that 10% and 11% of the magnetic monopods led to two-patch and three-patch PS particles, respectively (Figure 8b). These results are quite close to those observed with non-magnetic monopods (17% and 3%, respectively). Consequently, experiments—which remain to be performed—under conditions with at least a four-fold increase in concentration should make it possible to significantly improve the yield and obtain particles with higher magnetic patch number.

## 4. Conclusions

In the present study, we showed that the solvent-induced self-assembly route is efficient and robust for obtaining PS particles of a few hundred nanometers with several 100 nm silica or magnetic silica patches emerging at their surface. The magnetic silica patches are silica particles, which each encapsulate a single maghemite supraparticle. Thus, the proposed paradigm shift is realistic: it is possible to envision the fabrication of polymer particles with several patches by solvent-induced self-assembly of single-patch particles, which are usually easier to synthesize. We can nevertheless identify two avenues for improving both the process and the morphology of the as-obtained multipatch particles. Firstly, the process only makes it possible to obtain mixtures of particles with different numbers of patches. Sorting steps should, therefore, be considered to separate the particles with 2 patches, 3 patches, etc. This can be envisaged by density gradient centrifugation [34], or by sorting using a magnetic field gradient for particles with magnetic patches, as previously developed for narrowing size distribution in ferrofluids [35]. Secondly, it must be admitted that the particles with several patches are not more symmetrical than those reported elsewhere [16,17]. One undoubtedly effective way for the patches to fit into repulsion figures would be both to reduce the volume of the PS nodules on the monopods and/or to promote repulsive forces between the patches, for example, by grafting ionic groups on the surface of the patches.

When homogeneous batches of particles with several magnetic patches are obtained, then assembly studies under a magnetic field will be possible and will have to be correlated with studies by simulation integrating the exact geometry of these multipatch particles. We can nevertheless imagine that these particles with several inorganic patches, magnetic or not, will also be of interest in applications independently of any assembly, such as nanomotors, nanostirrers, nanoswimmers, e-paper pixels, targeted drug delivery and water treatment.

## Figures and Tables

**Table 1:** Morphological distribution and geometrical parameters of the silica/PS particles as a function of the nominal grafting surface density d_MPS_. Experimental conditions: [100-nm silica] = 7.3 × 10^15^ part./L; [styrene] = 89 g/L; [Synperonic^®^ NP30] = 2.85 g/L; [SDS] = 0.15 g/L; [Na_2_S_2_O_8_] = 0.46 g/L; 70 °C and 6 h. Representative TEM images are shown in Figure S1.

Entry	d_MPS_ (funct./nm^2^)	Monomer-to-Polymer Conversion (%)	Final Batch Composition (%) ^1^					PS Nodule Diameter (nm)	
			Monopods	Bipods	Tripods	Tetrapods	Multisilica ^2^	Monopods	Free Particles
#1.1	0.3	95	17	38	32	11	2	210	128
#1.2	0.5	88	63	29	6	-	2	214	126
#1.3	0.6	63	99	-	-	-	1	165	98
#1.4	0.7	60	98	-	-	-	2	163	94
#1.5	1.0	64	99	-	-	-	1	127	82
#1.6	2.0	70	99	-	-	-	1	145	113
#1.7	0.6	88	97	-	-	-	3	220	151
#1.8	0.6	68	98	1	-	-	1	148	102

^1^ Fractions expressed with respect to the silica seeds, i.e., free PS particles were not considered. ^2^ Multisilica are silica/PS particles made of two or more silica seeds.

**Table 2:** Final distribution of the number of silica patches on the PS particles as a function of the composition of the ethanol/THF mixture and the concentration of monopods. Experimental conditions: [monopods] = C = 7.3 × 10^14^ part./L at 25 °C for 24 h. Representative TEM images are shown in Figure S2 for entries #2.3 to #2.7.

Entry.	Ethanol/THF Volume Ratio	Monopod Concentration	Final Distribution of the Number of Silica Patches (%) ^1^				
			N = 1 ^2^	N = 2	N = 3	N = 4	N ≥ 5
#2.1	50/50	C	100	-	-	-	-
#2.2	40/60	C	100	-	-	-	-
#2.3	30/70	C	91	7	2	-	-
#2.4	20/80	C	80	17	3	-	-
#2.5	10/90	C	76	13	4	1	6
#2.6	20/80	2C	77	19	3	1	-
#2.7	20/80	4C	38	35	17	5	5

^1^ Expressed in fraction of monopods assembled in PS particles of similar patch number; ^2^ corresponds to the fraction of unassembled monopods.

**Table 3:** Morphological distribution and geometrical parameters of the magnetic silica/PS particles as a function of the nominal grafting surface density d_MPS_. Experimental conditions: [100-nm silica] = 4.8 × 10^14^ part./L; [styrene] = 56.8 g/L; [Synperonic^®^ NP30] = 0.19 g/L; [SDS] = 0.01 g/L; [Na_2_S_2_O_8_] = 0.78 g/L; 70 °C and 6 h. Representative TEM images are shown in Figure S4 for entries #3.1, #3.2 and #3.4.

Entry	d_MPS_ (funct./nm^2^)	Monomer-to-Polymer Conversion (%)	Final Batch Composition (%) ^1^							
			Monopods	Bipods	Tripods	Tetrapods	Pentapods	Hexapods	Other Multipods	Multisilica ^2^
#3.0 ^3^	0.6	95	96	-	-	-	-	-	-	4
#3.1	0.6	88	4	9	4	4	6	65	7	1
#3.2	1.0	76	3	6	14	30	10	23	11	3
#3.3	1.5	66	44	30	19	6	-	-	-	1
#3.4	1.9	93	90	1	-	-	-	-	-	9
#3.5	1.9	85	96	-	-	-	-	-	-	4
#3.6	1.9	91	94	2	-	-	-	-	-	4

^1^ Fractions expressed with respect to the magnetic silica seeds, i.e., free PS particles were not considered; ^2^ multisilica are magnetic silica/PS particles made of two or more seeds; ^3^ reference experiment carried out from pure silica seeds.

## Data Availability

The data presented in this study are available on request from the corresponding author.

## Supplementary Materials

The following are available online at https://www.mdpi.com/2079-4991/11/1/147/s1, Figure S1: Representative TEM images of silica/PS nanoparticles obtained for different nominal MPS grafting surface densities; Figure S2: Representative TEM images of silica/PS monopods after solvent-induced assembly as a function of the composition of the ethanol/THF mixture and the concentration of monopods; Figure S3: Silica strengthening and coating of the maghemite supraparticles with a graph showing the thickness shift, infrared spectrum and representative TEM image of maghemite supraparticles and size distribution; Figure S4: Representative TEM images of silica/PS monopods after centrifugal sorting.
