# Supplementary material for: Towards Polymeric Nanoparticles with Multiple Magnetic Patches
Source: Nanomaterials (Basel). 2021 Jan 9;11(1):147. doi: 10.3390/nano11010147 (PMC7827819; doi:10.3390/nano11010147)
Supplement: Supplementary file 1 [file nanomaterials-11-00147-s001.pdf]

nanomaterials

Article

Towards Polymeric Nanoparticles with Multiple Magnetic Patches

Elham Yammine [1,2], Laurent Adumeau [1], Maher Abboud [3], Stéphane Mornet [1], Michel Nakhl [2] and Etienne Duguet [1,*]

[1] Univ. Bordeaux, CNRS, Bordeaux INP, ICMCB, UMR 5026, 33600 Pessac, France; elhamyammine93@gmail.com (E.Y.); laurent.adumeau@cbni.ucd.ie (L.A.); stephane.mornet@icmcb.cnrs.fr (S.M.)
[2] LCPM/PR2N (EDST), Lebanese University, Faculty of Sciences II, 90656 Jdeidet El Metn, Lebanon; mnakhl@ul.edu.lb
[3] Unité Environnement Génomique et Protéomique, U-EGP, Faculté des Sciences, Université Saint-Joseph, Campus des Sciences et Technologies Mar Roukos–B.P. 1514, Riad El Solh, Beirut 1107 2050, Lebanon; maher.abboud@usj.edu.lb
* Correspondence: etienne.duguet@icmcb.cnrs.fr; Tel.: +33-540-002-651

Abstract: Fabricating future materials by self-assembly of nano-building blocks programmed to generate specific lattices is among the most challenging goals of nanotechnology and has led to the recent concept of patchy particles. We report here a simple strategy to fabricate polystyrene nanoparticles with several silica patches based on the solvent-induced self-assembly of silica/polystyrene monopods. The latter are obtained with morphological yields as high as 99% by seed-growth emulsion polymerization of styrene in the presence of 100 nm silica seeds previously modified with an optimal surface density of methacryloxymethyl groups. In addition, we fabricate "magnetic" silica seeds by silica encapsulation of preformed maghemite supraparticles. The polystyrene pod, i.e., surface nodule, serves as a sticky point when the monopods are incubated in a bad/good solvent mixture for polystyrene, e.g., ethanol/tetrahydrofuran mixtures. After self-assembly, mixtures of particles with two, three, four silica or magnetic silica patches are mainly obtained. The influence of experimental parameters such as the ethanol/tetrahydrofuran volume ratio, monopod concentration and incubation time is studied. Further developments would consist of obtaining pure batches by centrifugal sorting and optimizing the relative position of the patches in conventional repulsion figures.

Keywords: silica; polystyrene; maghemite supraparticles; patchy particles; seeded-growth emulsion polymerization; solvent-induced self-assembly

Citation: Yammine, E.; Adumeau, L.; Abboud, M.; Mornet, S.; Nakhl, M.; Duguet, E. Towards Polymeric Nanoparticles with Multiple Magnetic Patches. *Nanomaterials* **2021**, *11*, 147. https://doi.org/10.3390/nano11010147

Received: 21 December 2020
Accepted: 6 January 2021
Published: 9 January 2021

Publisher's Note: MDPI stays neutral with regard to jurisdictional claims in published maps and institutional affiliations.

Copyright: © 2021 by the authors. Submitted for possible open access publication under the terms and conditions of the Creative Commons Attribution (CC BY) license (http://creativecommons.org/licenses/by/4.0/).

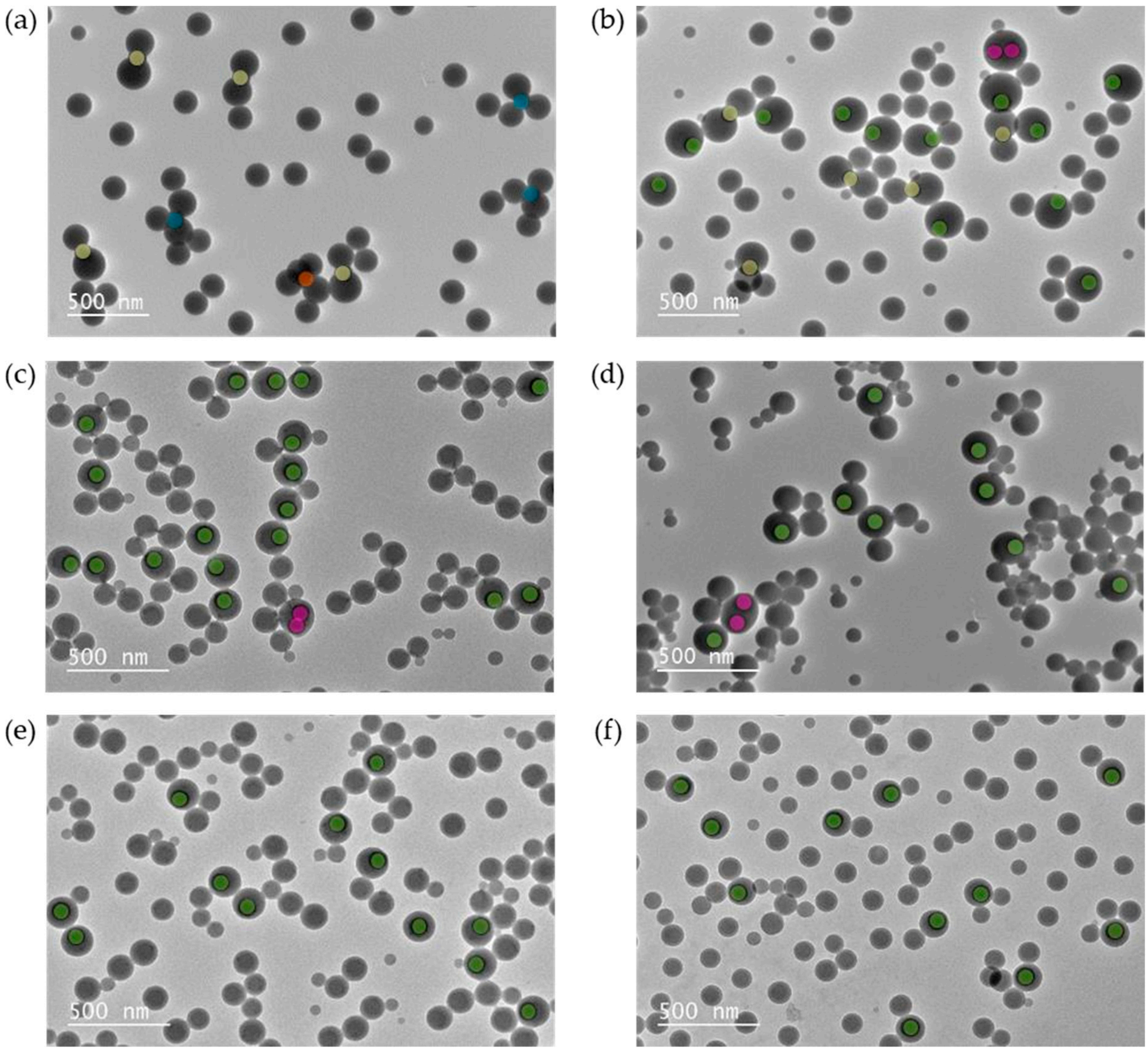

Figure S1. Representative TEM images of silica/PS nanoparticles obtained for different nominal grafting surface densities d_{MPS}: (**a**) 0.3 funct./nm^2; (**b**) 0.5 funct./nm^2; (**c**) 0.6 funct./nm^2; (**d**) 0.7 funct./nm^2; (**e**) 1 funct./nm^2 and (**f**) 2 funct./nm^2. The silica cores are falsely colored in green for monopods, yellow for bipods, blue for tripods, orange for tetrapods and magenta for multi-silica particles. Results of statistical analyses are presented in Table 1 (Entries #1.1 to #1.6). Free PS particles are usually removed by centrifugation, but they were kept in this experiment in order to present the whole result of the polymerization step.

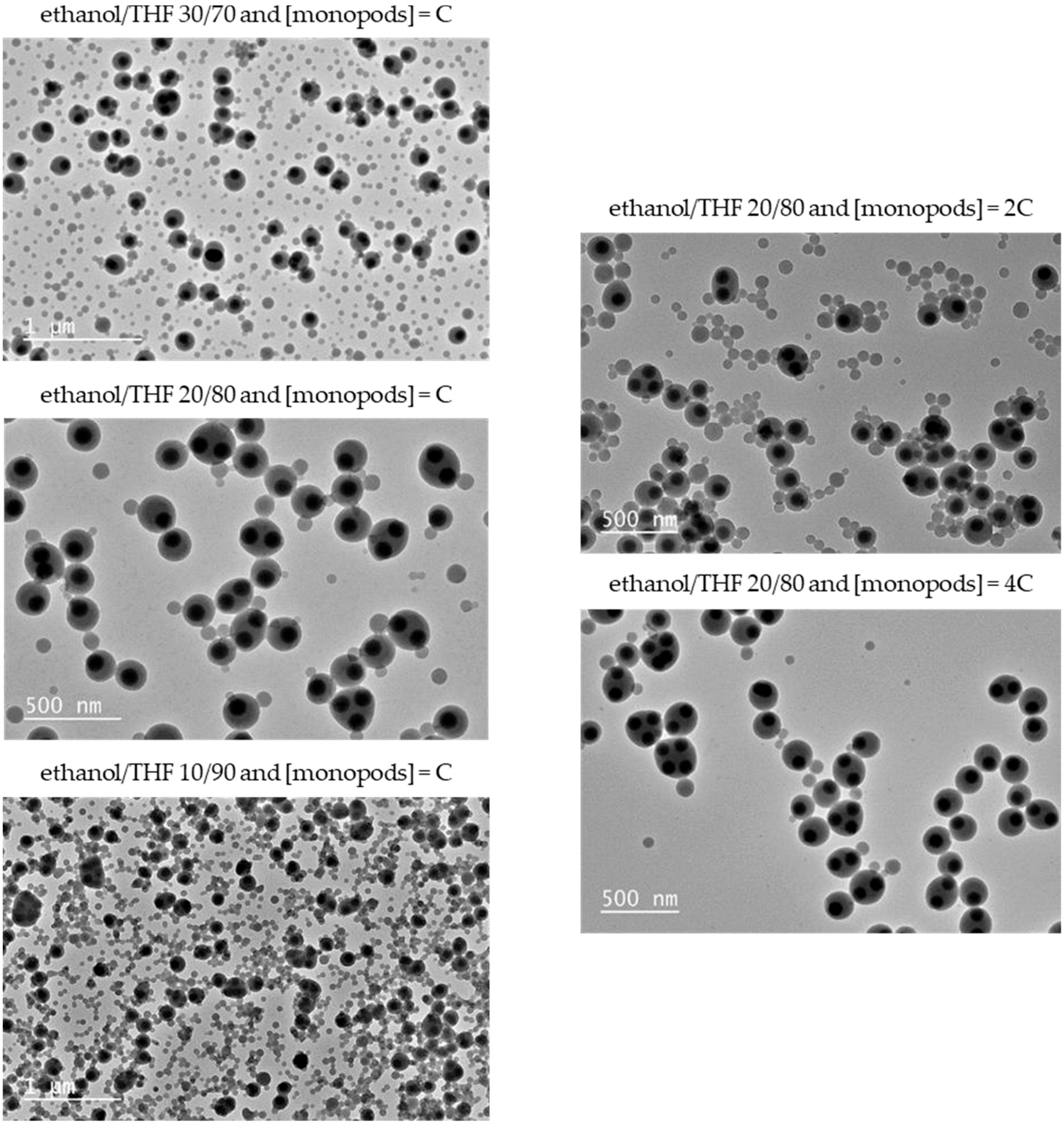

Figure S2. Representative TEM images of silica/PS monopods (Table 1, entry #1.3) after solvent-induced assembly as a function of the composition of the ethanol/THF mixture and the concentration of monopods (Table 2, entries #2.3 to #2.7). Experimental conditions: [monopods] = C = 7.3×10^{14} part./L at 25 °C for 24 h.

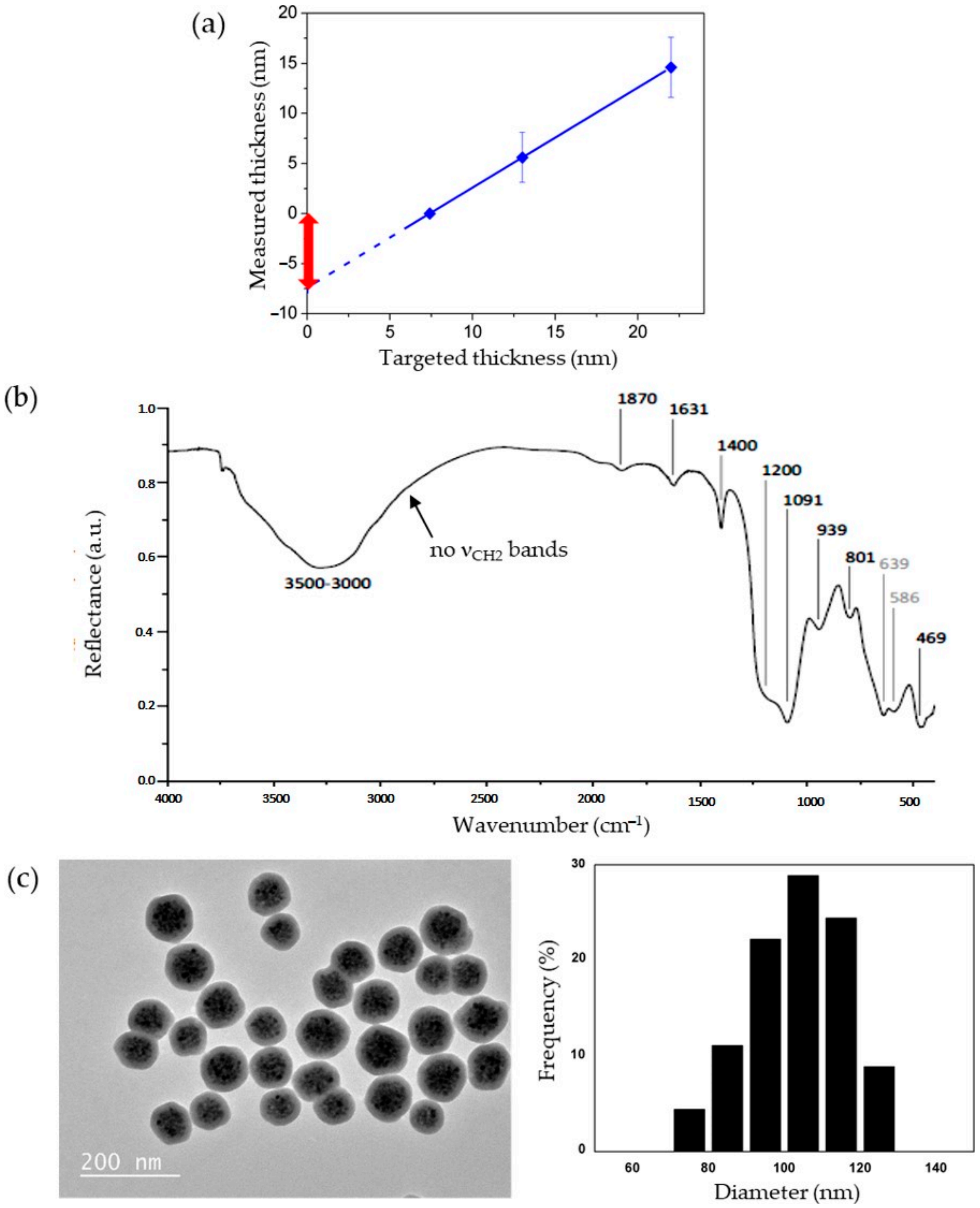

Figure S3. Silica strengthening and coating of the maghemite supraparticles: (**a**) graph showing the systematic shift of about 8 nm between the measured thickness and the targeted thickness of the silica coating demonstrating that part of the TEOS used served to fill the spaces between the maghemite nanoparticles within the supraparticles, (**b**) infrared spectrum of silica-coated maghemite supraparticles showing that stearic acid and SDS are no longer present, and (**c**) TEM image of maghemite supraparticles with 13-nm silica coating and size distribution as determined by statistical analysis of TEM images. Diffuse reflectance infrared Fourier transform spectroscopy was performed with a Bruker IFS Equinox 55 FTIR (Palaiseau, France) spectrometer (signal averaging 128 scans at a resolution of 4 cm^{-1}) equipped by a selector Graseby Specac diffuse reflection cell (Eurolabo, Paris, France). A volume of the nanoparticle dispersion was dried at 80 °C under vacuum to obtain the sample in powder form. Then, samples were prepared by spreading crushed powders (3 wt.%) in anhydrous (spectroscopy grade) KBr on a conical support.

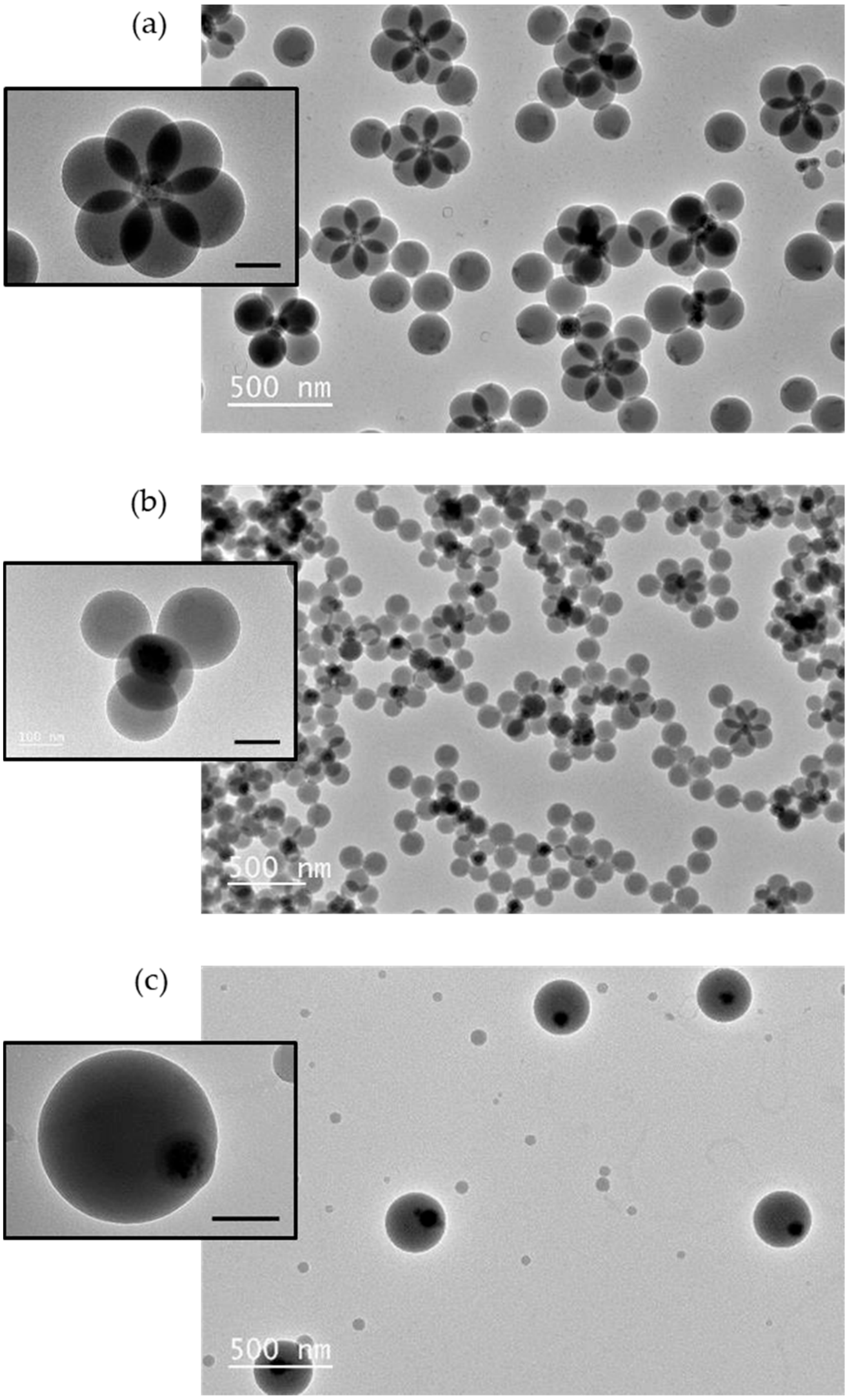

Figure S4. Representative TEM images of magnetic silica/PS particles obtained after seed-growth emulsion polymerization of styrene in the presence of silica-coated maghemite supraparticles surface-modified with MPS at different nominal grafting densities: (**a**) 0.6 funct./nm^2; (**b**) 1 funct./nm^2 and (**c**) 1.9 funct./nm^2. They correspond to entries #3.1, #3.2 and #3.4, respectively (Table 3). Scale bars: 500 nm and 100 nm for insets.
